# Clinical Cases

**Published:** 1893-04

**Authors:** G. Pompili

**Affiliations:** Rome, Italy


### INTERNATIONAL HAHNEMANNIAN ASSOCIATION MEETING OF 1892.

### CLINICAL CASES.

G. Pompili, M. D., Rome, Italy.

Case I.—The family of Mr. A. L. F., of Boston, lived in Rome for many years. On December 22d, 1876, I was called to attend Mrs. F., who was seriously ill, and had been for more than six months, from the effects of a cold which had produced much intestinal disturbance, resulting in an obstinate diarrhœa. There had been also a suppression of an eruption (acne rosaceæ) which had covered the face for several years. I believe that a single dose of Dulcamara, given at the time, would have brought back the normal condition, and made a cure. But the family physician was a mongrel, one of those self-styled homœopaths, who, not valuing the instructions of Hahnemann in all their minute parts, lose the mariner's compass and fail to find the similar remedy. So, many other remedies were administered, but without effect, till the hot season coming on, the family left Rome. The Roman physician recommended a physician from Naples, another mongrel, who continued the same treatment without having any regard to the precepts of *The Organon*, and the principal remedies were Scorza ris parinio (?) in low dilutions and Calc-iod., second and third triturations. These were wholly ineffectual, and the poor invalid returned to Rome in October in worse condition than when she left, and under the former doctor the old methods continued, the patient always growing worse. As I have stated, I was called December 22d, 1876. I found her in a truly pitiable state, which seemed to give little hope of life: Pale, emaciated, with rapid pulse, great thirst, and sunken eyes, and prostration to the degree that movement was impossible without the aid of two attendants to help her. The necessity of frequent change of position, especially at night, and the distress which the great emaciation gave her in whatever position she could be placed, gave her almost no opportunity to sleep. The chief characteristic expression of the affection was found in the intestinal evacuations, neither liquid or watery, but constituted one mass, in part cylindrical, in part pultaceous, of matter all undigested and smeared with blood. The food, which consisted almost entirely of meat, was evacuated almost as it was swallowed, and appeared like meat wrapped up in light, bloody mucus. With a mental glance at our materia medica, and without great consideration, my mind fixed upon Phos., and I gave this remedy for three successive mornings. December 22d, 23d, 24th, a dose of five globules of the 2c each morning. A decided improvement appeared. All the symptoms were alleviated. The evacuations began gradually to occur regularly, and strength by degrees to return. And here I wish to note a gratifying coincidence which confirmed my selection of the remedy. Before I

was called to Mrs. F. her husband sent to our excellent colleague, Dr. W. P. Wesselhœft, who had been his family physician in Boston, a statement of the malady which affected his wife. The latter was full two weeks in reaching Boston. Dr. Wesselhœft telegraphed the remedy, but in the meantime improvement had begun, as stated above. Now the remedy which Dr. Wesselhœft mentioned in the telegram was Phosph. The remedies which I gave afterward, as noted in my register, are Dulc.$^{2c}$, Sulph.$^{2c}$, Lach.$^{2c}$, Phos.$^{6m}$ (Jenichen), Ambra$^{2m}$, and for the last Psorin$^{2c}$, an interval of twenty or thirty days elapsing between remedies.

The recovery was complete, and Mrs. F. saved from certain death, by the first of June, and on returning to Rome in October found herself better able to breathe the air of the Campagnia than before her sickness. After some years the family returned to the United States and Mrs. F. still lives in good health.

Case II.—Gabrielle Lubowidzka, Polish, and a Sister of the Sacred Family of Nazareth—one of those many institutions by means of which Christian charity diffuses among the people the culture and the example of every virtue. The child of parents of a most unhappy temperament, both of whom died in early life of phthisis, as well as an older sister, with still another sister (younger) affected with the same disease, came under my care in 1885. She had suffered a long time from a throat trouble, was much debilitated, and there was a prospect of a near end. She coughed very frequently, expectorated yellow, putrid matter, of salt taste. There was fever, preceded by chills in the afternoon, terminating always with perspiration. There was great emaciation and exhaustion, voice weak and hoarse, and eyes sunken. The menses were suppressed, and there were many other symptoms not noted because of no importance in this connection. I have decided to report the case because of a circumstance which occurred during the second year of my treatment of the case.

In March, 1886, Dr. Leon Simon, of Paris, came to Rome for a few days. The Superior of the Sisters, who had met him in her travels, and had lived near him in Paris, desired me to bring him to visit the invalid Sister, Gabrielle. In my company Dr. Simon visited the patient, examined her, thoroughly approved of my treatment, but gave his opinion, which was also that of all the religious community, that the poor girl had only five or six months to live.

I have the faculty of almost never losing hope. The power of Homœopathy, all the marvelous cures of which there are so many testimonials, inspires me always with confidence, which is sometimes too great. I continued the treatment, which, after another year and one-half, carried the invalid to recovery to such an extent that Sister Gabrielle was sent as Superior to Coracoria, where she remained for more than three years, enduring the severe winters of that northern climate. Afterward she passed a winter in Paris, where Dr. Leon Simon was able to see the invalid, whose restoration to health he had thought impossible, no longer weak and emaciated, but vigorous and well nourished.

A long time was required to complete this cure, in part because of my absence from Rome during the summer season. It was lengthened to more than three years. I will note in chronological order the medicines used, as I find them in my register: Calc-c$^{2c}$, Merc.$^{2c}$, Puls.$^{2c}$, Bell.$^{2c}$, Natr-mur.$^{2c}$, Phos.$^{6m}$ (Jenichen), China$^{2c}$, Sil.$^{2c}$, Bell.$^{2c}$, China$^{2c}$, Bry.$^{cm}$ (Skinner, during 1885), Dulc.$^{cm}$ (Fincke), Kali-carb.$^{cm}$ (F.), Merc.$^{6m}$ (F.), Nux-vom.$^{50m}$ (F.), Bell.$^{40m}$ (F.), Ars.$^{40m}$ (Jenichen and C. M. Fincke), Ars.$^{40m}$ (Jen.), Hepar$^{cm}$ (F.), Merc.$^{cm}$ (F.), Dulc.$^{cm}$ (F.), Phos.$^{20m}$ (F.), Hydr-ac$^{30}$, Merc.$^{6m}$ (F. in 1886), Lyc.$^{cm}$ (F.), Bell.$^{40m}$ (F.), Sep.$^{2c}$, Spong.$^{2c}$, Colocynth.$^{cm}$ (F.), Sil.$^{72m}$ (F.), Baryt-carb.$^{2c}$, Ars.$^{cm}$ (Swan in 1887), Cinnabar$^{cm}$ (F.), Phos.$^{70m}$ (F.), Hepar$^{cm}$ (F. in 1888). These remedies were administered at long or short intervals, according to the exigencies of the case, sometimes after a few days, oftener after one month.

*Remarks.*—These two cases show me, above all, that disease holds out much more obstinately, and demands much longer time and greater patience to cure when the origin of the patients is from parents unhealthy and affected with chronic miasm, as was discovered and illustrated by Hahnemann. The two ladies who furnished these clinical histories were eminently psoric, and of unhappy constitutions. Enough to say that the first had erysipelatous eruptions permanently on the face, and the second nearly died of pulmonary phthisis, of which both parents and a sister had died. In regard to the administration of remedies it should be observed that in the first case very little of the first remedy was required to produce a profound impression upon an organism already saturated with improper medicines; all the other remedies were given in the single dose. The greatest and most difficult question is this of repetitions, which, unless properly made, may hinder the cure. We have fundamental instruction from Hahnemann, it is true, upon this point, but it is also true that "it is original sin not to know how to wait," as Gross has expressed it. The illustrious Dr. Fincke has lately written: "This subject, therefore, is so vast that it demands time and patience to collect from it the laws and rules of the posology of Homœopathy."

One single canon or law appears, however, to be established with certainty, and in accordance with it we worked in the cases given above, and continue to work in similar cases; for example, when we treat persons exhausted and with little or weak reaction or vital-force, we ought always to give the single dose. Lastly, we wish to make two brief remarks for the encouragement of the true friends of Homœopathy:

First, our experience in the first case brings out the fact that our law of cure—the law of nature—guarantees, when well applied, this harmony of prescriptions which never in the world any other school of medicine has known how to bring about, and that to the one scientific system of medicine which it was reserved to Hahnemann to discover. So that, from one part of the world to the other, as in this case—from Rome to Boston, from Constantinople to Lisbon, from Algiers to Pekin—given the same symptoms, the same prescription will follow.

The second remark refers to the second case, which shows and proves the superiority of Hahnemannian Homœopathy to routine Homœopathy, that now is more followed. Also, Dr. Leon Simon, an excellent man and doctor of medicine, whose father was truly

Hahnemannian, ought to throw his influence against the preponderance of that mongrel sect inaugurated by Tessier which is responsible in great part for the adulteration of Homœopathy in France, where, in general, *The Organon* is as if it did not exist, and where habitually only the 4th, 6th, and 12th dilutions are used, while we hold that without the high and highest potencies, and without the strictest observance of the precepts of *The Organon*, this cure could not have been obtained. Honor to the great Master, and to all International Hahnemannians who defend and make triumphant his teachings!

